# Supplementary material for: Salicylic Acid Mediates Chitosan-Induced Immune Responses and Growth Enhancement in Barley
Source: Int J Mol Sci. 2024 Dec 10;25(24):13244. doi: 10.3390/ijms252413244 (PMC11676727; doi:10.3390/ijms252413244)
Supplement: Supplementary file 1 [file ijms-25-13244-s001.zip › ijms-3357000-supplementary.pdf]

Supplementary

Table S1. List of primers used for RNAseq gene expression verification and *F. graminearum* and barley gDNA quantification.

	Gene name	Gene full name	Gene ID	Orientation	Primer sequence
RNAseq verification	*ARF*	*ADP-Ribosylation Factor*	AJ508228	Forward	GCTCTCCAACAACATTGCCAAC
				Reverse	GCTTCTGCCTGTCACATACGC
	ACT	*Actin*	AK362208.1	Forward	AAGTACAGTGTCTGGATTGGAGGG
				Reverse	TCGCAACTTAGAAGCACTTCCG
	NPR1	*Nonexpresser of PR genes1*	AM050559	Forward	CCAAAACAGTTGAACTCGGCAA
				Reverse	GACGATGAGGAAGCTGAAAGGGTTG
	PR9	*Pathogenesis related gene9*	CAA41294	Forward	CTCCTGTGCCGACATACTCA
				Reverse	GTCCGTGTTTGCCTCATTCT
	PR4	*Pathogenesis related gene4*	BAK04328	Forward	CTGTCGTGGCGGAGCAAGTA
				Reverse	ATCCCGTTGGTGTCGATCTTG
	PR14	*Pathogenesis related gene14*	CAA48621.1	Forward	TGAGCCCCTGCATCTCCTAT
				Reverse	CGCAGGCATAGCTGAGTGTA
F. graminearum and barley gDNA quantification	*Hv_EFG1*	*Translation elongation factorG1*	AY836205.1	Forward	CGGGCATCTAGGATTCAATAAC
				Reverse	AGCAAAGCCAGTTAAATTCTCG
	Fg_TRI5	*trichodiene synthase5*	FGSG_03537	Forward	GARCAGTACAACTTTGGAGG
				Reverse	ACCATCCAGTTCTCCATCTG

Table S2. List of differentially expressed genes mentioned in the manuscript.

Gene name	Assembly R2 Gene ID
ICS	HORVU.MOREX.r2.5HG0393770.1
PAL1	HORVU.MOREX.r2.2HG0150540.1
PAL2	HORVU.MOREX.r2.6HG0494410.1
PAL3	HORVU.MOREX.r2.6HG0494420.1
NPR1	HORVU.MOREX.r2.3HG0196030.1
NPR3	HORVU.MOREX.r2.3HG0242450.1
NPR4	HORVU.MOREX.r2.4HG0278140.1
WRKY6	HORVU.MOREX.r2.7HG0584730.1
WRKY33	HORVU.MOREX.R2.4HG0316370.1
WRKY70	HORVU.MOREX.r2.7HG0591390.1
PR1	HORVU.MOREX.r2.5HG0392230.1
PR2	HORVU.MOREX.r2.3HG0266280.1
PR3	HORVU.MOREX.R2.1HG0043210.1
PR4	HORVU.MOREX.R2.3HG0272550.1
PR5	HORVU.MOREX.r2.UnG0626650.1
PR6	HORVU.MOREX.R2.1HG0055720.1
PR7	HORVU.MOREX.R2.4HG0344400.1
PR9	HORVU.MOREX.r2.2HG0092440.1
PR10	HORVU.MOREX.r2.5HG0367890.1
PR14	HORVU.MOREX.r2.3HG0188660.1
PR15	HORVU.MOREX.r2.4HG0279610.1
PR16	HORVU.MOREX.r2.4HG0349160.1

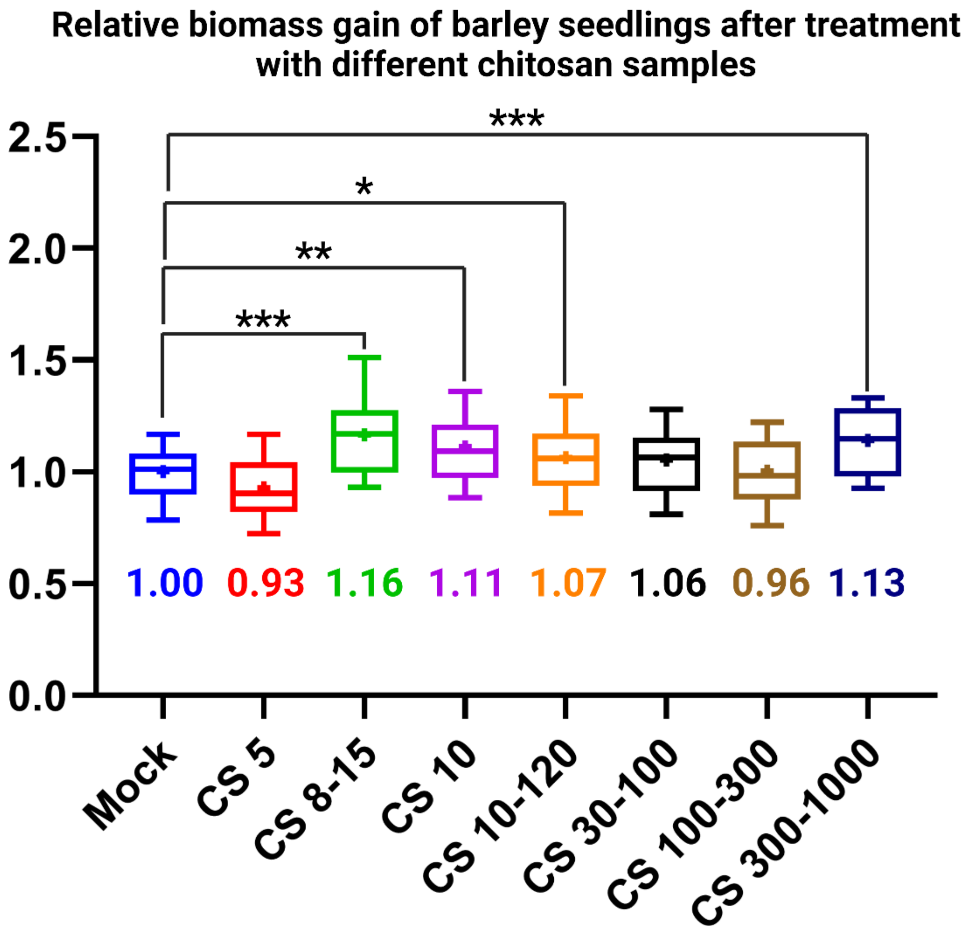

Figure S1. Relative biomass gain of barley seedlings after 19 days of cultivation in Hoagland medium after treatment with respective chitosan samples (each in concentration of 200 ppm). Each box represents the percentile in range 25-75; the whiskers represent the percentile 10 and 90. Asterisks indicate significance level (based on one-way ANOVA and Tukey's post hoc test) * $p \le 0.05$, ** $p \le 0.01$ and *** $p \le 0.001$.
